# Human *FOXN1*-Deficiency Is Associated with αβ Double-Negative and FoxP3+ T-Cell Expansions That Are Distinctly Modulated upon Thymic Transplantation

**DOI:** 10.1371/journal.pone.0037042

**Published:** 2012-05-10

**Authors:** Adriana S. Albuquerque, José G. Marques, Susana L. Silva, Dario Ligeiro, Blythe H. Devlin, Jacques Dutrieux, Rémi Cheynier, Claudio Pignata, Rui M. M. Victorino, M. Louise Markert, Ana E. Sousa

**Affiliations:** 1 Instituto de Medicina Molecular, Faculdade de Medicina, Universidade de Lisboa, Lisboa, Portugal; 2 Hospital de Santa Maria, Centro Hospitalar Lisboa Norte, Lisboa, Portugal; 3 Immunogenetics Laboratory, Centro de Histocompatibilidade do Sul – CHSul, Lisboa, Portugal; 4 Division of Allergy and Immunology, Department of Pediatrics, Duke University Medical Center, Durham, North Carolina, United States of America; 5 Institut Cochin, Département Immunologie-Hematologie, Paris, France; 6 Inserm, U567, Paris, France; 7 Université Paris Descartes, Faculté de Médecine René Descartes, UMR-S 8104, Paris, France; 8 Pediatric Immunology Unit, Department of Pediatrics, “Federico II” University, Naples, Italy; 9 Department of Immunology, Duke University Medical Center, Durham, North Carolina, United States of America; 10 CNRS, UMR 8104, Paris, France; New York University, United States of America

## Abstract

Forkhead box N1 (FOXN1) is a transcription factor crucial for thymic epithelium development and prevention of its involution. Investigation of a patient with a rare homozygous FOXN1 mutation (R255X), leading to alopecia universalis and thymus aplasia, unexpectedly revealed non-maternal circulating T-cells, and, strikingly, large numbers of aberrant double-negative αβ T-cells (CD4negCD8neg, DN) and regulatory-like T-cells. These data raise the possibility that a thymic rudiment persisted, allowing T-cell development, albeit with disturbances in positive/negative selection, as suggested by DN and FoxP3+ cell expansions. Although regulatory-like T-cell numbers normalized following HLA-mismatched thymic transplantation, the αβDN subset persisted 5 years post-transplantation. Involution of thymus allograft likely occurred 3 years post-transplantation based on sj/βTREC ratio, which estimates intrathymic precursor T-cell divisions and, consequently, thymic explant output. Nevertheless, functional immune-competence was sustained, providing new insights for the design of immunological reconstitution strategies based on thymic transplantation, with potential applications in other clinical settings.

## Introduction

The thymus is a primary lymphoid organ essential for normal T-cell development. The unique ability of the thymic microenvironment to generate and select T-cells requires a specialized epithelium that is regulated by forkhead box N1 (FOXN1) [1]–[4]. This transcription factor is expressed by the thymic anlage that emanates from the epithelium of the pharyngeal pouch, and is required for the differentiation of thymic epithelial cells [1], [2]. Thymic epithelium comprises two main compartments defined by their position and functional characteristics: the cortex, and the medulla, the latter being thought to be fundamental for the negative selection of auto-reactive thymocytes and the generation of central tolerance. The cortical and medullary epithelia have been shown, in mice, to differentiate from a common epithelial progenitor that expresses *FOXN1* [4]. Additionally, continuous *FOXN1* expression was shown to be essential for both the maintenance of thymopoiesis in the murine postnatal period [4], and the prevention of thymic involution during adulthood [3], [5], highlighting FOXN1 as an important target for immune reconstitution strategies. Mutations in *FOXN1* lead to athymia together with total alopecia, due to the additional role of FOXN1 in hair follicle differentiation [1], [2], [6].

Human *FOXN1*-deficiency was first reported by Pignata *et al.* in two sisters from Campania, Italy [7], [8]. Notwithstanding the evidence of athymia, a significant number of circulating T-cells was observed in these children with close to normal numbers of CD8 T-cells [9]. We identified the same homozygous R255X mutation [7], [9], [10], in a Portuguese child, who presented at 5 months with total alopecia and Bacillus Calmette-Guérin (BCG) dissemination, following routine neonatal vaccination with this live-attenuated mycobacterium, that also presented with a significant pool of circulating non-maternal T-cells [11]. This child underwent HLA mismatched thymic transplantation with evidence of functional immunologic reconstitution, as we recently reported [11].

The aim of this work was to investigate the T-cell compartment of this *FOXN1*-deficient child and the mechanisms underlying the immunological reconstitution upon thymic transplantation. Our findings suggest that T-cell development can occur in a putative *FOXN1*-deficient thymus rudiment, albeit with altered positive and negative selection as suggested by the marked expansion of T-cells expressing the alpha-beta T-cell receptor (TCR) in the absence of both CD4 and CD8 expression (double-negative DNαβ) together with an over-representation of T-cells of a regulatory-like phenotype expressing high levels of FoxP3 in conjunction with other regulatory markers. Notwithstanding, an extrathymic origin of the altered T-cell populations cannot be discarded. We also showed that the transplantation of HLA-mismatched FOXN1 competent thymic epithelium led to the achievement of sustained immune-competence despite evidence of involution of the allogeneic thymus 3 years post-transplantation. We believe that these data on the follow-up of this unique clinical case contribute significantly to the debate on the mechanisms underlying T-cell development and immune reconstitution that may help in the design of new therapeutic approaches in other clinical settings.

**Figure 1:**
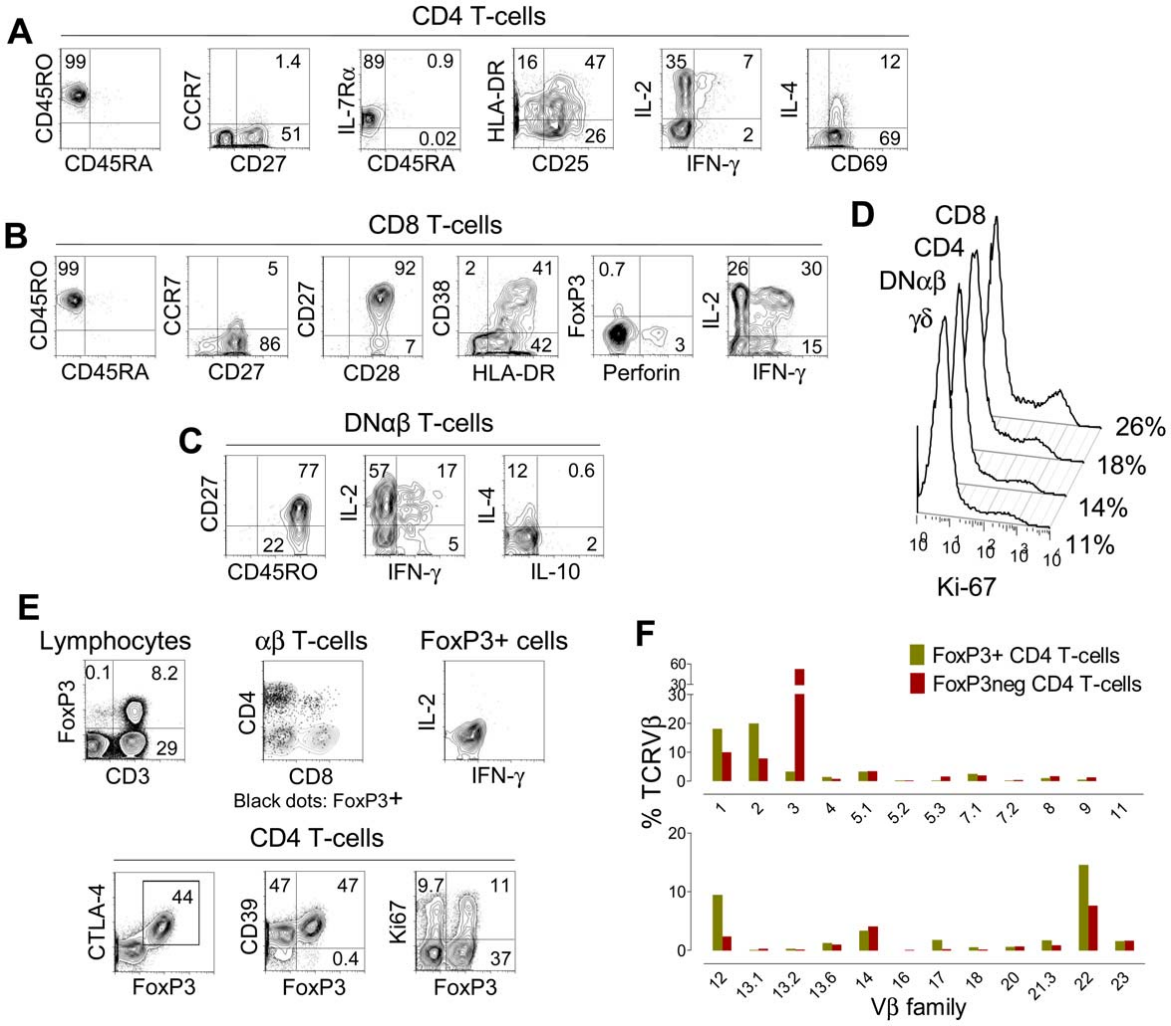
Peripheral T-cells prior to thymus transplantation in a patient with R255X *FOXN1* mutation. At day 166 of life there were 10.4% (660 cells/µl) CD4 T-cells, 9.7% (612 cells/µl) CD8 T-cells; and 10.7% (676 cells/µl) DNαβ T-cells in the peripheral blood. CD4+ (**A**), CD8+ (**B**), and DNαβ T-cells (**C**) exhibited a memory-effector activated phenotype and were able to produce significant amounts of IL-2, IFN-γ and IL-4. (**D**) Percentages of cycling cells, as assessed by Ki-67 expression, within CD8, CD4, DNαβ and γδ T-cells. (**E**) Dot-plots showing that: FoxP3 expression is restricted to CD3+ cells; that within total FoxP3+ cells (328 cells/µl) there were 67% CD4+, 17% DNαβ and 12% cells co-expressing CD4 and CD8 (DP, DP represented 3% of total αβ cells and 26% of them were FoxP3+) as illustrated by the expression of CD4 and CD8 within total αβ cells (grey) and within FoxP3+ cells (black); the concomitant expression of FoxP3 with other Treg markers (CTLA-4, CD39), or Ki-67 within total CD4+ T-cells; and the lack of cytokine production by FoxP3+ cells. (**F**) Vβ distribution within FoxP3+ and FoxP3− CD4 T-cells performed at day 254 of life. Graphs show the proportion of FoxP3+ CD4 T-cells and FoxP3− CD4 T-cells belonging to a given Vβ family as assessed by flow cytometry. Dot-plots show analysis after gating on the respective populations; numbers within each quadrant represent the proportion of cells expressing the respective molecules. Cytokine production was assessed after 4 hours stimulation of PBMC with PMA plus Ionomycin in the presence of Brefeldin A.

**Figure 2:**
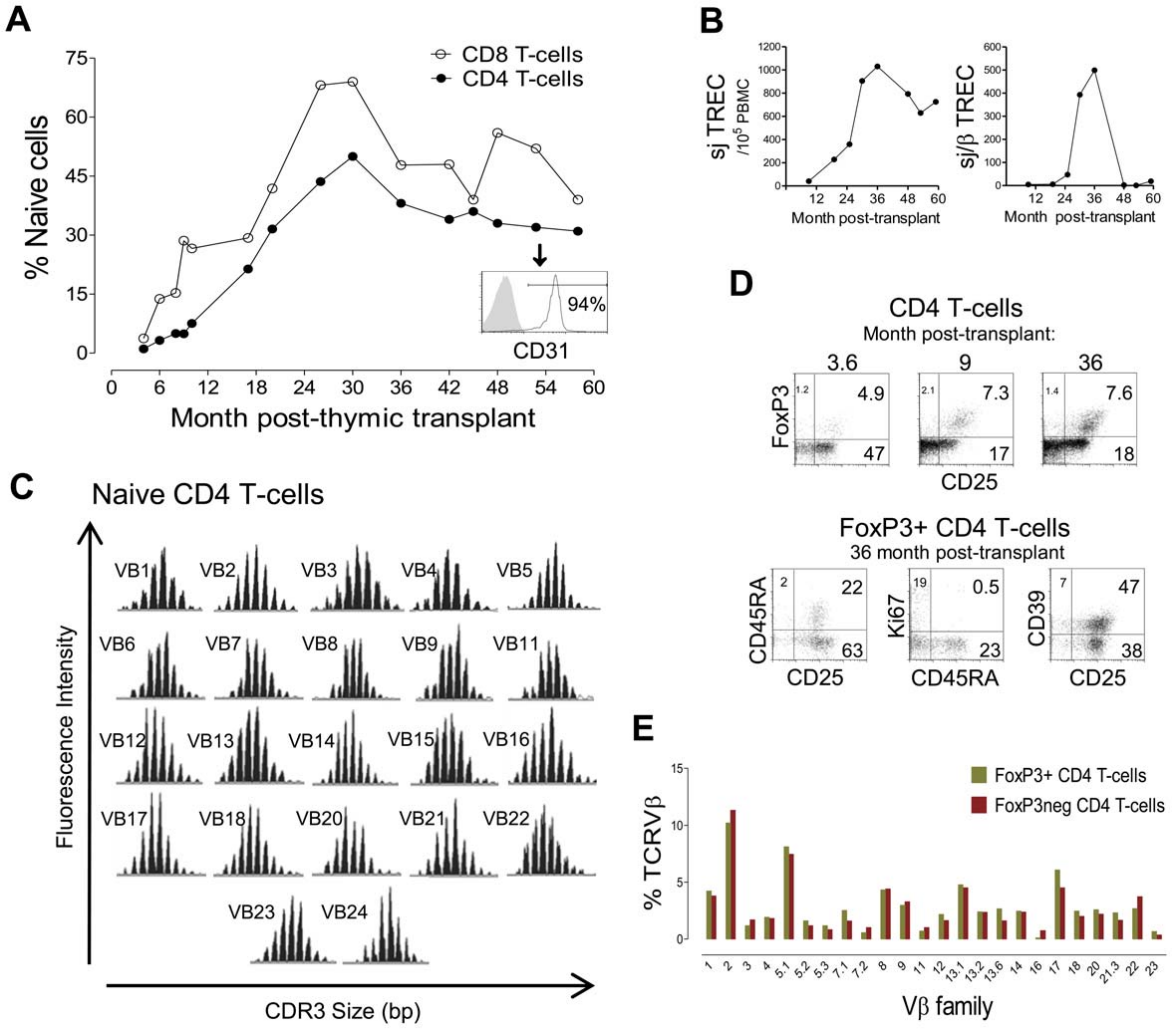
Immunological reconstitution and Treg recovery after thymic transplantation in a patient with R255X *FOXN1* mutation. (**A**) Kinetics of the frequency of naïve cells (CD45RA+CD27+) within CD4 and CD8 T-cell subsets; histogram shows CD31 expression within naïve CD4 T-cells at 58 months post-transplant, a marker associated with recent thymic emigrants. (**B**) Longitudinal quantification of sjTREC (left) and sj/βTREC ratio (right) quantified in total PBMC. (**C**) Assessment of TCR repertoire by spectratyping analysis of the CDR3 Vβ regions of purified naïve CD4 T-cells at 59 months post-transplant. (**D**) Representative dot-plots of the longitudinal analysis of the frequency of cells expressing FoxP3 and/or CD25 within total CD4 T-cells (upper dot-plots), and the phenotype of circulating FoxP3+ cells at 36 months post-transplant after successive gates on CD4+ and FoxP3+ T-cells (lower dot-plots); numbers inside quadrants represent the frequency of cells expressing the indicated molecules. (**E**) Graph shows the proportion of circulating FoxP3+ and FoxP3− CD4 T-cells belonging to each of the Vβ families at 48 months post-transplantation analysed by flow cytometry.

## Methods

### Patient

Female child, born at term to consanguineous Portuguese parents, admitted at day 157 of life with respiratory failure due to Bacillus Calmette-Guérin (BCG) dissemination, following routine neonatal BCG vaccination. The *FOXN1* mutation identified is a homozygous C-to-T transition at nucleotide position 792 (GenBank accession no. Y11739) leading to a nonsense mutation at residue 255 (R255X) in exon 4, formerly exon 5 [10]. Maternal chimerism was assessed using AmpFISTR Identifiler PCR Amplification Kit (Applied Biosystems, detection limit 1/100). The patient's clinical data were the focus of another manuscript [11]. Failure to thrive and progressive nutritional status deterioration were observed despite antibiotic/tuberculostatic therapy and intravenous immunoglobulin G. Thymus transplantation was performed (day 424 of life), under protocols approved by the Duke Institutional Review Board (IRB) and reviewed by the Food and Drug Administration under an Investigational New Drug (IND) application, as described [11]. Unrelated allogeneic thymus tissue, routinely discarded from infants less than 9 months of age undergoing cardiac surgery, was used for transplantation after informed consent [12]–[14].

**Figure 3:**
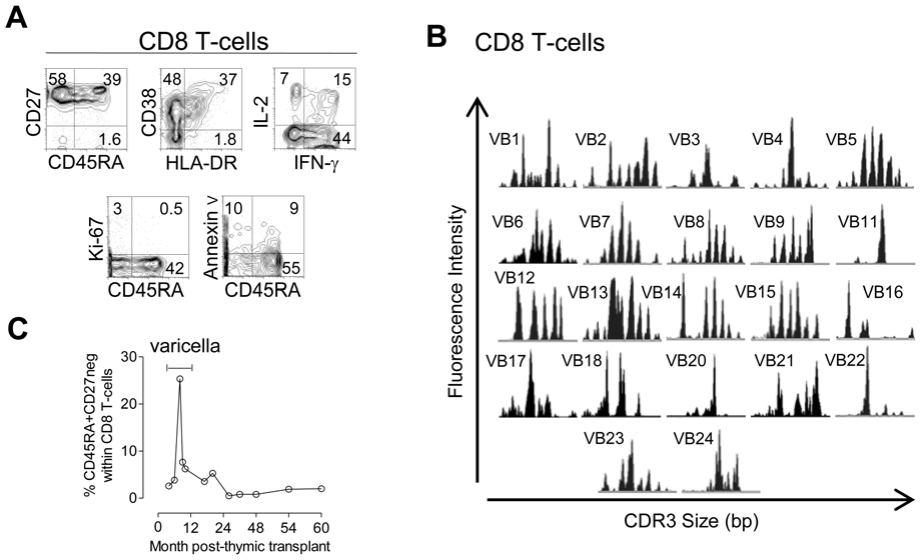
Reduced but functional CD8+ T-cell compartment after fully-mismatched HLA class I thymus transplantation. (A) Circulating CD8+ αβ T-cells analysed 58 months post-transplantation (5% of total lymphocytes; 83cells/µl) by flow cytometry in terms of: naïve/memory/effector phenotype assessed by CD45RA and CD27 expression; levels of activation markers (CD38 and HLA-DR); IFN-γ and IL-2 production upon PMA+ionomycin stimulation; *ex vivo* frequency of cycling cells (Ki-67+); and *ex vivo* frequency of apoptotic cells assessed by annexin V staining. (B) TCR repertoire diversity evaluated by spectratype of CDR3 Vβ regions of purified CD8+ T-cells at 59 months post-transplant. (C) Graph shows the coordinate increase in the proportion of terminally differentiated effector cells defined as CD45RA+CD27neg cells within total CD8+ T-cells during acute varicella infection and its decrease in parallel with its clinical resolution.

### Ethics

Studies were performed with parent's written informed consent under the ethical guidelines of Hospital Universitário de Santa Maria, Faculdade de Medicina da Universidade de Lisboa, and the Duke University Health Systems. The Ethical Board of the Hospital Universitário de Santa Maria, Faculdade de Medicina da Universidade de Lisboa specifically approved this study.

### Cell isolation and cell sorting

Peripheral blood mononuclear cells (PBMC) were isolated immediately after collection by Ficoll-Hypaque. Naïve CD4+ T-cells were sorted using the EasySep CD4+ naïve T-cell enrichment magnetic kit (StemCell Technologies). DNαβ and CD8 T-cells were isolated using the BD FACSAria High Speed Cell Sorter (BD Biosciences) after surface staining for CD3, CD4, CD8, TCRγδ and TCRαβ. Population purity after sorting was greater than 98%.

**Figure 4:**
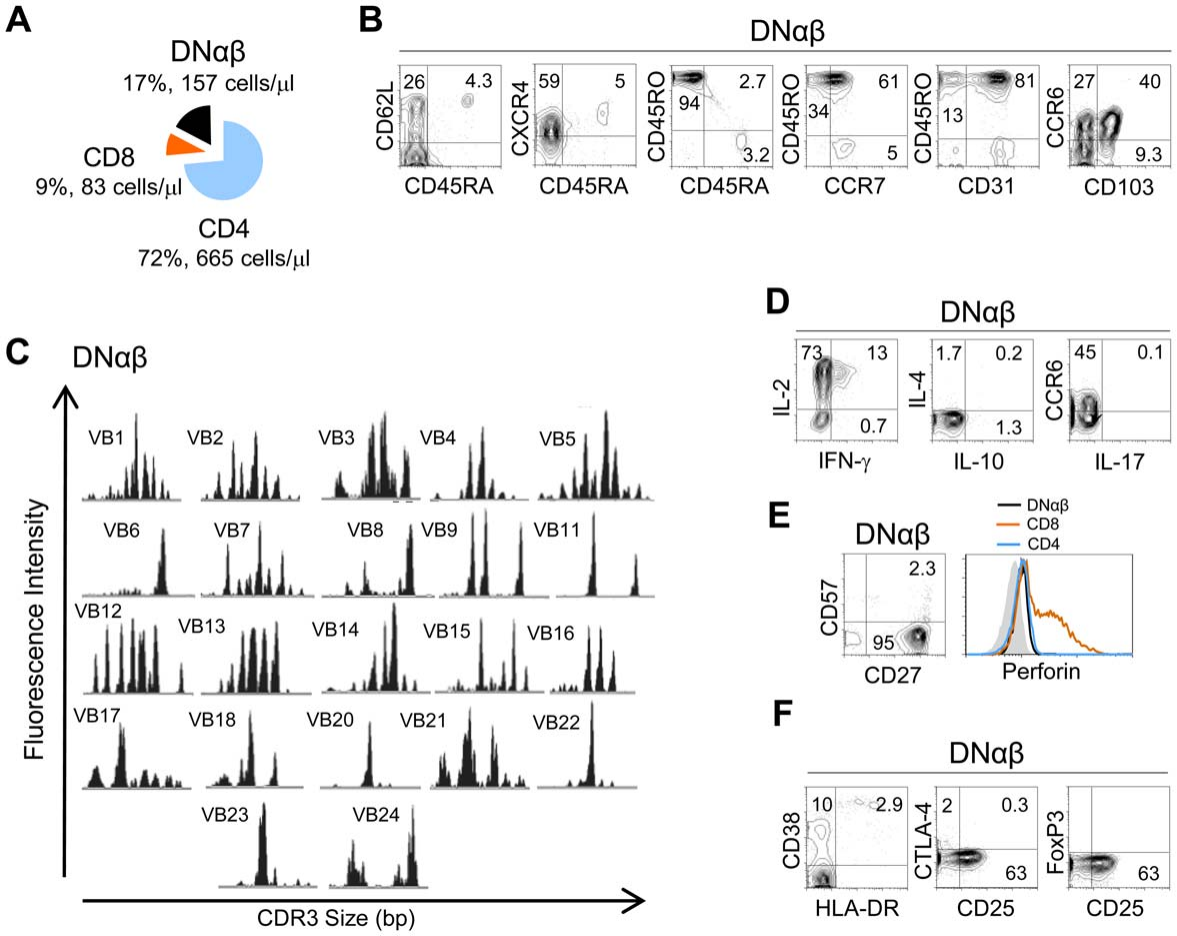
Persistence of DNαβ T-cells in a patient with R255X *FOXN1* mutation after thymic transplantation. (**A**) Absolute counts and proportion of DN, CD8+, CD4+ cells within circulating αβ+ T-cells 59 months post-transplantation. Analysis of DNαβ T-cells revealed: (**B**) a relatively undifferentiated memory phenotype with increased expression of mucosal homing molecules; (**C**) a skewed repertoire as assessed by spectratype of CDR3 Vβ regions; (**D**) reduced ability to produce IFN-γ, IL-4, or IL-17 but high IL-2 production upon PMA+ionomycin stimulation; (**E**) no terminal-effector differentiation according to CD27, CD57 and perforin expression (histogram compares perforin levels within DNαβ, CD8 and CD4 T-cells); (**F**) reduced levels of the activation markers CD38 and HLA-DR and absence of Treg-associated markers despite the increased levels CD25 expression, Numbers inside dot-plots represent frequency of cells expressing the indicated molecules acquired with FACSCalibur flow cytometer.

### Flow Cytometry

Lymphocyte subsets were characterized using fresh whole blood after acquiring at least 100,000 events within a lymphogate using a FACSCalibur flow cytometer (BD Biosciences). TCR Vβ family frequency was quantified in whole blood using IOTest Beta Mark (Beckman Coulter). PBMC were stained intracellularly for CTLA-4 (clone BNI3) and/or Ki-67 (clone MOPC-21) both from BD Biosciences, and FoxP3 (clone PCH101) using eBiosciences's kit after surface staining, as described [15]. FoxP3 expression post-transplant was assessed in fresh PBMC, whereas cryopreserved PBMC were used for time-points prior to thymic transplantation. Apoptosis was assessed in fresh PBMC using Annexin V-FITC Apoptosis Detection Kit (BD Biosciences) and propidium iodide (PI) staining. Analysis was done using FlowJo software (TreeStar). Results were expressed as median intensity of fluorescence (MFI) of a molecule or percentage of positive cells, and absolute numbers calculated by multiplying their percentage by the absolute lymphocyte count.

**Figure 5:**
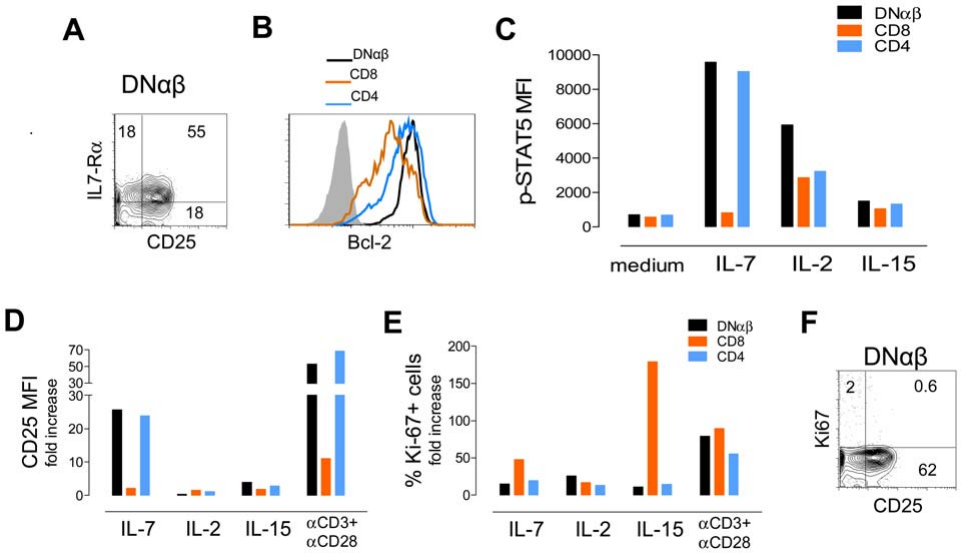
Ability of expanded circulating DNαβ T-cells to respond to IL-7 and IL-2. Representative flow cytometric analysis of freshly isolated PBMC from a patient with R255X *FOXN1* mutation 59 months after thymic transplantation illustrating (**A**) the preserved expression of IL-7Rα and increased CD25 expression within DNαβ T-cells; and (**B**) the high levels of Bcl-2 expression within DNαβ in comparison with CD8 and CD4 T-cells. (**C**) Up-regulation of p-STAT5 upon 15 min stimulation of PBMC with IL-7 (50 ng/ml), IL-2 (100 U/mL) or IL-15 (25 ng/ml), bars represent p-STAT5 MFI within gated DNαβ, CD8 and CD4 T-cells. Freshly isolated PBMC were cultured for 5-day in the presence of IL-7 (10 ng/ml), IL-2 (10 U/mL), or IL-15 (12.5 ng/ml) or anti-CD3 plus anti-CD28 stimulation and graphs represent the fold change of CD25MFI with respect to medium (**D**), and the frequency of Ki-67+ cells (**E**), within gated DNαβ, CD8 and CD4 T-cells. (**F**) Representative analysis of freshly isolated PBMC illustrating the low levels cycling cells (Ki-67+) despite the increased CD25 expression within gated DNαβ T-cells. Numbers inside dot-plots represent frequency of cells expressing the indicated molecules acquired with FACSCanto (p-STAT5 and 5-day cultures) or FACSCalibur flow cytometers.

### Assessment of cytokine production at the single-cell level

Cytokine production was assessed at single-cell level after 4 h PBMC culture with PMA/ionomycin in the presence of brefeldin A, as described [16], with mAb against IFN-γ (clone 4S.B3), IL-2 (clone MQ1-17H12), and IL-4 (clone MP4-25D2) from BD Biosciences; IL-10 (clone JES3-9D7); and IL-17 (clone eBio64DEC17) from eBiosciences.

### STAT5 tyrosine phosphorylation analysis

STAT-5 phosphorylation (p-STAT5) was evaluated by flow cytometry on fresh PBMC stimulated for 15 min with IL-7 (50 ng/ml), IL-2 (100 U/mL) or IL-15 (25 ng/ml) or medium alone as described [17].

### Proliferative responses to cytokines

Fresh PBMC were stimulated with IL-7 (10 ng/ml), IL-2 (10 U/mL), IL-15 (12.5 ng/ml), immobilized anti-CD3 (1 µg/ml) plus anti-CD28 (1 µg/ml) or medium for 5 d. The fold change in percentage of Ki-67+ cells and CD25 MFI with respect to medium alone were evaluated by flow cytometry on gated DNαβ, CD4+ and CD8+ T-cells.

### TCR – chain CDR3 spectratyping

Total RNA was extracted from 10^5^ to 10^6^ cells with RNeasy kit (Qiagen) and first strand cDNA synthesized from 1–2 µg of RNA with the Superscript III kit (Invitrogen) using an equivolume mixture of random hexamers and oligo (dT). Amplification of the TCRVβ CDR3 was performed using primers specific for each Vβ family [18] except for Vβ6 and Vβ21 [19] and a common Cβ reverse primer [18]; followed by a run-off reaction that extends each different PCR product with a constant Cβ FAM labeled primer; and a third step, in which each different Vβ PCR labeled fragment was separated using a capillary electrophoresis based DNA automated sequencer. Data were collected and analyzed with GeneMapper v4.0 (Applied Biosystems) for size and fluorescence intensity determination.

### TREC analysis

Signal joint (sj) and DβJβ T-cell receptor rearrangement excision circle (TREC) analyses were conducted as described [20], [21]. Briefly, triplicate multiplex PCR amplification for sjTREC, DJβ1TRECs (Dβ1-Jβ1.1 to 1.6) or DJβ2TRECs (Dβ2-Jβ2.1 to 2.7), together with the CD3γ chain were performed on lysed PBMC. TREC and CD3γ quantifications were then performed using a LightCycler™ in independent experiments, with the same first-round serial dilution standard curve. This highly sensitive nested quantitative PCR assay allowed detection of one copy in 10^5^ cells for any excision circle. The sj/βTREC ratio, (sjTREC/10^5^cells/(DJβ1TRECs/10^5^cells+DJβ2TRECs/10^5^cells)), was calculated as described [20].

## Results

### Abnormal expansion of DNαβ and FoxP3^bright^ T-cells in human FOXN1-deficiency

Circulating T-cells of non-maternal origin were documented at close to normal numbers (2219 cells/µl) at 5 months of age in a child with a homozygous R255X mutation in the *FOXN*1 gene, as we recently reported [11]. The T-cell compartment exhibited similar proportions of CD4+, CD8+ and, αβ-cells that expressed neither CD4 nor CD8 (double-negative, DNαβ), which usually represent less than 1% of circulating T-cells. Athymia was diagnosed based on absent thymus-shadow on x-ray, lack of naïve T-cells, and undetectable levels of products of TCR rearrangements in the thymus, specifically signal-joint TCR excision circles (sjTREC) [11]. Here, we aimed to further investigate the phenotype and the function of these T-cells developed in the context of a putative athymia. We found that CD4+ T-cells exhibited an activated memory-effector phenotype with preserved IL-2, IFN-γ and IL-4 production (Figure 1A). CD8+ T-cells featured a similar activated phenotype, with no terminal-effector differentiation, as illustrated by the maintenance of CD45RO/CD28/CD27 expression (Figure 1B) and the low frequency (3%) of perforin-producing cells (Figure 1B). The aberrantly expanded DNαβ T-cells (676 cells/µl) also expressed CD45RO, most were CD27+, and produced IL-2 (Figure 1C), in agreement with a lack of terminal-effector differentiation [17]. A significant proportion of T-cells were cycling (Figure 1D). It is likely that IL-7 played a role in T-cell maintenance/expansion given their preserved expression of the IL-7 receptor α-chain (IL-7Rα), as illustrated for CD4 T cells in Fig. 1A, and lack of elevated serum IL-7 levels (6.3 pg/ml, 209 days pre-transplantation). IL-7 serum levels typically increase in lymphopenic settings [17]. In agreement with the evidence of IL-7 use, IL-7 serum levels were not increased before transplantation, and increased transitorily to 44.3 pg/ml (133 days post-transplantation) during T-cell depletion in the peri-transplantation period.

The thymus is known to produce a regulatory CD4+ T-cell subset (Treg), fundamental for preventing autoimmunity, currently best identified by expression of the forkhead box P3 transcription factor (FoxP3) [22]–[25]. We found that up to 40% of the CD4 subset (328 cells/µl) expressed high levels of FoxP3, and observed atypical populations of FoxP3+ DNαβ and double-positive T-cells (Figure 1E). FoxP3 can also be up-regulated in non-Treg T-cells upon activation [24], [26]. Nonetheless, several findings support these cells being *bona fide* Treg. In agreement with human Treg phenotype [24], they expressed FoxP3 at high intensity concomitantly with other Treg-associated markers, namely CTLA-4 and CD39 (Figure 1E). Moreover, in contrast to activated T-cells, they did not produce IL-2 or IFN-γ (Figure 1E). The limited amount of blood samples precluded suppressive assays as well as the evaluation of Treg-like cell repertoire by CDR3 sequence analysis. Of note, comparison of the relative representation of different Vβ families within the FoxP3+ and FoxP3− CD4 sub-populations revealed a skewed repertoire in both populations but with distinct oligoclonal expansions in each, providing further support that these FoxP3+ cells represented a separate CD4 lineage (Figure 1F).

Thus, despite the presence of *FOXN1*-deficiency, we observed a reasonable number of circulating non-maternal T-cells associated with oligoclonal expansions of DNαβ and FoxP3+ T-cells. The presence of these cells suggested the persistence of a thymic rudiment supporting a perturbed T-cell development, or, alternatively, that extrathymic sites of lymphopoiesis could be involved in T-cell development in the context of a congenital absence of the thymus.

### Sustained immune-competence was achieved upon HLA mismatched thymic transplantation despite involution of thymus allograft

As *FOXN1* mutations impact on thymic epithelium rather than on hematopoietic precursors, we predicted that thymic transplantation, although never previously performed in this setting, could provide a curative strategy. As shown in our previous clinical report [11], the efficacy of the thymic transplantation performed was best demonstrated by the temporal association between the clearance of ongoing BCG adenitis and the development of PPD-specific proliferative responses. We continued to follow the child and confirmed that she remained free of significant infections 5 years after cessation of all prophylactic therapies. Here, we aimed to further investigate the kinetics of T-cell recovery upon HLA-mismatched thymic transplantation and its relationship with output of the thymic explants.

A slow progressive increase in the proportion of circulating naïve CD4+ T-cells, mainly expressing CD31, a marker associated with recent-thymic emigrants [17], was observed (Figure 2A), accompanied, as expected, by an increase in sjTREC levels within total PBMC (Figure 2B). Despite the HLA-mismatch of the thymic epithelia, naïve CD4+ T-cells showed a fully diverse TCR repertoire 5 years post-transplantation (Figure 2C), confirming the trend shown in our previous report within total CD4 T-cells [11].

The expanded Treg-like cells disappeared during the peri-transplant period and a parallel reconstitution of the Treg pool and CD4 subset was observed, leading to stable frequencies within the normal range (Figure 2D) as well as absolute counts (44 cells/µl, 36 months post transplantation). Of note, distribution of Vβ-families within FoxP3+ and FoxP3− CD4+ T-cells was very similar despite the HLA-mismatch between thymic epithelia and host (Figure 2E).

CD8+ T-cell recovery was disproportionally low compared to CD4 T-cells (92 cells/µl, 9% of T-cells, 5 years post-transplantation), confirming our previous report [11]. Nevertheless, we show here that the kinetics of naïve cell expansion within CD8+ subset paralleled those observed for their CD4+ counterparts (Figure 2A). In agreement, we found similar sjTREC levels in purified CD4+ and CD8+ T-cells (7155 and 7540 sjTREC/10^5^ cells; respectively, 30 months post-transplantation). A poor CD8 recovery has been described following HLA-mismatched thymic transplantation in DiGeorge syndrome [12], [13], possibly related to the full HLA class I mismatch between host hematopoietic precursors and allogeneic thymic epithelia or to alterations in the thymic graft associated with transplantation procedures. Nevertheless, the transplant-derived CD8+ T-cells were apparently functional in this child [11], as documented by the transitory expansion of terminally differentiated CD8 T cells during Varicella Zoster virus infection (Figure 3).

Additionally, in order to estimate the functionality of the allogeneic thymic graft we quantified, for the first time in the context of thymic transplantation, the sj/βTREC; a ratio between early and late products of TCR rearrangements providing an indirect measurement of thymocyte division-rate and a direct correlate of thymic output [20], [27], [28]. Whilst very low during the peri-transplant period, the sj/βTREC ratio increased progressively (Figure 2B), reaching levels comparable to those observed in healthy children, 2.5 years post-transplant. Of note, a sharp decline in sj/βTREC was observed 4 years post-transplantation (Figure 2B). This was accompanied by a modest decrease in sjTREC levels within total PBMC and in the proportion of naïve cells, supporting the decline in thymic allograft output (Figure 2A–B). These values plateaued thereafter (Figure 2A–B), suggesting that a steady-state equilibrium was established after replenishment of the immune system.

These data provide novel evidence that immune-competence can be achieved in the absence of long-term sustainability of thymic output from the allogeneic tissue, with implications for other clinical settings aimed at immunological reconstitution.

### Persistence of circulating DNαβ T-cells despite the immunological recovery upon thymic transplantation

We also investigated the fate of the DNαβ T-cell population that was markedly expanded pre-transplantation [11]. In contrast with the recovery of all T-cell subsets, a significant population of circulating DNαβ persisted, at relatively stable numbers (range 125–548 cells/µl throughout the follow-up) [11], being 188 cells/µl at 6-years post-transplantation (Figure 4A).

DNαβ cells maintained a similar memory phenotype, being all CD45RO+ (Figure 4B), and skewed repertoire, as assessed by spectratyping (Figure 4C), throughout the follow-up period. They also displayed no evidence of terminal-effector differentiation as indicated by their preserved ability to produce IL-2 in the absence of significant amounts of effector cytokines such as IFNγ, IL-4, IL-10 or IL-17, following short-term PMA/Ionomycin stimulation (Figure 4D), as well by their preserved expression of CD27, in the absence of CD57 and perforin (Figure 4E). A potential ability for mucosal homing was suggested by significant expression of CCR6 and CD103 (Figure 4B), which was of even more interest given that DNαβ cells neither produced IL-17 (Figure 4D) nor expressed the Treg markers FoxP3 or CTLA-4 (Figure 4F). Their levels of CD38 and HLA-DR expression were low (Figure 4F), suggesting that they were not in an activated state.

Importantly, DNαβ cells expressed high levels of CD25, the IL-2 receptor α-chain, in conjunction with CD127, the IL-7 receptor α-chain (Figure 5A). Moreover, they showed higher levels of Bcl-2 expression than CD4 and CD8 T-cells (Figure 5B), suggesting *in vivo* responsiveness to IL-7. To test this hypothesis we quantified the levels of STAT-5 phosphorylation (p-STAT-5) within DNαβ, CD4 and CD8 T-cells upon short-term exposure to IL-7, IL-2 or IL-15 *in vitro*. As shown in Figure 5C, DNαβ cells exhibited the highest levels of p-STAT-5 upon IL-2 or IL-7 stimulation and were not responsive to IL-15. Additionally, freshly isolated PBMC were cultured in the presence and in the absence of these cytokines as well with anti-CD3 plus anti-CD28 mAb as a positive control for T-cell stimulation. We found that, as for CD4 T-cells, DNαβ cells up-regulated CD25 expression upon IL-7 stimulation *in vitro* (Figure 5D), but showed no distinct proliferative abilities, as assessed by the expression of the cell-cycling marker Ki67, in response to these cytokines in comparison with CD4 or CD8 T-cells (Figure 5E). Of note, they respond to TCR stimulation with both up-regulation of CD25 (Figure 5D) and increased frequency of cycling cells (Figure 5E). Notably, the frequency of cycling DNαβ cells *ex vivo* was very low (Figure 5F). Thus, our findings supported a role of IL-7 and IL-2 in DNαβ cell maintenance, given their high levels of expression of IL-7 and IL-2 receptors and ability to respond to these cytokines *in vitro*. Moreover, DNαβ cells showed increased Bcl-2 levels in the absence of cell cycling markers *in vitro* and *ex vivo*, suggesting that IL-7 and/or IL-2 mainly impacted on DNαβ cell survival rather than their turnover.

In order to evaluate the thymic contribution for the maintenance of DNαβ cells, we quantified the sjTREC levels in sorted DNαβ cells from the peripheral blood 6 years post thymus transplantation. The sjTREC levels were found to be very low (3.2 sjTREC/10^5^ DNαβ cells as compared with 3783 sjTREC/10^5^ sorted CD4 T cells and with 2212 sjTREC/10^5^ sorted CD8 T cells), supporting the possibility that DNαβ were long-lived cells originated before the thymus transplantation.

Overall, we found that the DNαβ cells abnormally expanded before thymic transplantation were maintained for up to six years post-thymic transplantation and appeared to rely mainly on cytokine-driven survival.

## Discussion

The transcription factor *FOXN1* is considered essential for the development of the thymic epithelia and the prevention of its involution during adulthood. Nevertheless, we demonstrated here that human homozygous R255X *FOXN*1-deficiency may be associated with significant numbers of circulating T-cells of non-maternal origin with major expansions of DNαβ and FoxP3+ cells through the study of a rare clinical case.

Our data raise important questions regarding T-cell origin in the context of human athymia. One plausible explanation is that a thymic rudiment may persist, facilitating a limited production of T-cells that subsequently expanded in the periphery. In mice, the *Foxn1* gene was shown to be dispensable for the initial formation of the thymic primordium [4], [29]. There is also evidence of functional T-cells in nude mice [30], although, at least some of these T-cells seem to be generated extra-thymically [31]. CD4+ and CD8+ αβ T-cells accumulate with ageing in nude mice [32], but characterization of a putative Treg compartment has not been conducted. We have shown that FoxP3 induction can occur in early stages of both murine and human normal T-cell differentiation [15], [25]. Of note, significant DNαβ as well as FoxP3+ cells were found in a mouse model of extra-thymic lymphopoiesis induced by Oncostatin M, a cytokine that induces thymic atrophy and lymph node alterations that support T-cell differentiation [33].

Importantly, circulating T cells of non-maternal origin were found in all the cases described with R255X *FOXN1* mutation [7]–[9], [11], which leads to a short N-terminal FOXN1 protein without DNA binding domain [34]. In contrast no circulating T-cells were found in a patient with a second mutation identified in the human *FOXN1* gene (R320W), a missense mutation of the DNA binding domain [11]. These discrepant findings raise new hypotheses about the role of FOXN1 in the development of the thymic epithelium that deserve further exploration using mouse models and comparative structural studies [1], [35], [36].

As all patients reported with R255X *FOXN1* mutation presented circulating T-cells [7]–[9], [11], it is plausible that they retained a dysplastic thymic rudiment capable of supporting T-cell differentiation, albeit with a narrow TCR repertoire and impaired T-cell selection, allowing the emergence of atypical DNαβ and Treg. Alternatively, this thymic rudiment could allow T-cell commitment of precursors for subsequent extra-thymic development. In support of this possibility, progenitor T-cell commitment was shown to occur in the thymus prior to their extra-thymic development in mouse models [37].

The Treg compartment has recently been investigated in other clinical settings associated with peripheral oligoclonal T-cell proliferation in patients with thymic impairment either due to hypomorphic mutations in hematopoietic precursors (Omenn syndrome) [38]–[40], or to developmental defects associated with variable degrees of thymic hypoplasia (DiGeorge syndrome) [41], [42]. In these settings, Treg frequencies appeared to be unaltered or reduced [38]–[42], emphasizing the distinctiveness of the pattern of high levels of Treg seen before transplantation in our case of R255X *FOXN1*-deficiency. Remarkably, the peripheral pool of FoxP3+ T cells normalized upon transplantation of FOXN1 competent thymic epithelia. Thymic Treg development is currently thought to be dependent on a small developmental niche that tightly controls Treg output [43], [44]. It is thus plausible to speculate that FOXN1 may play a role in such niches, contributing to the thymic regulation of Treg numbers.

The numbers of circulating DNαβ T-cells remained relatively constant up to 6 years after thymic transplantation. Of note, these cells have not been shown to expand following HLA-mismatched thymus transplantation [11], [12], and were reported to progressively decline after effective naïve reconstitution in complete DiGeorge patients that presented with this atypical phenotype before thymic transplantation [12].

Regarding the origin of DNαβ T-cells, their reduced sjTREC levels suggest that they were not produced after transplantation. Moreover, it is unlikely that they are activated terminally-differentiated CD8+ T-cells that lost CD8 expression, as suggested in other clinical settings associated with abnormal expansions of circulating DNαβ cells, such as autoimmune lymphoproliferative syndromes (ALPS) [45], given the lack of expression of markers of terminal differentiation. The high levels of expression of mucosal-homing markers suggest an extra-thymic origin of DNαβ T-cells. They may have been generated pre-transplant or result from continuous *de novo* production. In the latter case their persistence 5 years post-transplantation favors a thymic rather than an extra-thymic origin since any putative extra-thymic lymphopoiesis is likely to be shut-down upon thymic transplantation [31]. We showed that DNαβ T-cells expressed high levels of IL-2 and IL-7 receptor α-chain and of Bcl-2 suggesting *in vivo* responsiveness to IL-7. Our data showing their ability to phosphorylate STAT-5 upon IL-7 or IL-2 stimulation further support this hypothesis. On the other hand, the low *ex vivo* frequency of cycling cells, and their reduced proliferative response to these cytokines, suggest that the DNαβ cell maintenance may be largely dependent on cytokine-induced survival, rather than cytokine-driven expansion.

In order to estimate the functionality of the allogeneic thymic graft, we took advantage sj/βTREC ratio quantification for the first time in this context. We found a progressive sj/βTREC increase in our clinical case, reaching levels comparable to those observed in healthy children. Importantly, a sharp decline of sj/βTREC, accompanied by a decrease in sjTREC levels and the proportion of naïve cells was observed 4 yrs post-transplantation. These values plateaued thereafter, illustrating the contribution of peripheral homeostasis to T cell pool maintenance following its replenishment, even in the absence of sustained thymic production. It is possible that the apparent lack of sustained thymocyte-division rate (as evidenced by sj/βTREC) resulted from an intrinsic reduced longevity of the thymus allograft or that the shut-down of the thymocyte production being a consequence of the replenishment of the peripheral pool. Given the rarity of *FOXN1* deficiency, the underlying factors contributing for the sustainability of the thymus allograft could be investigated in future studies on immunological reconstitution upon thymus transplantation in cases of DiGeorge syndrome with athymia. The data generated will be important to appraise the promising use of thymus transplantation in other clinical contexts.

Overall, our finding of significant numbers of oligoclonal T-cells in this case of *FOXN1-*deficiency due to R255X mutation suggest that, to a certain extent, T-cell development still occurs, albeit with altered positive/negative selection, as illustrated by the aberrant expansion of FoxP3+ and DN subsets. Importantly, we showed that immune-competence can be achieved through HLA-mismatched thymic transplantation, in spite of the apparent lack of long-term functionality of the allogeneic thymic tissue, which has potential implications for the design of immunological reconstitution strategies in other clinical settings.
